# Social determinants of dental treatment needs in Brazilian adults

**DOI:** 10.1186/1471-2458-14-1097

**Published:** 2014-10-23

**Authors:** Angelo Giuseppe Roncalli, Georgios Tsakos, Aubrey Sheiham, Georgia Costa de Souza, Richard G Watt

**Affiliations:** Department of Dentistry, Federal University of Rio Grande do Norte, Natal, RN Brazil; Department of Epidemiology and Public Health, University College London, London, UK

## Abstract

**Background:**

The chronic cumulative nature of caries makes treatment needs a severe problem in adults. Despite the fact that oral diseases occur in social contexts, there are few studies using multilevel analyses focusing on treatment needs. Thus, considering the importance of context in explaining oral health related inequalities, this study aims to evaluate the social determinants of dental treatment needs in 35–44 year old Brazilian adults, assessing whether inequalities in needs are expressed at individual and contextual levels.

**Methods:**

The dependent variables were based on the prevalence of normative dental treatment needs in adults: (a) restorative treatment; (b) tooth extraction and (c) prosthetic treatment. The independent variables at first level were household income, formal education level, sex and race. At second level, income, sanitation, infrastructure and house conditions. The city-level variables were the Human Development Index (HDI) and indicators related to health services. Exploratory analysis was performed evaluating the effect of each level through calculating Prevalence Ratios (PR). In addition, a three-level multilevel modelling was constructed for all outcomes to verify the effect of individual characteristics and also the influence of context.

**Results:**

In relation to the need for restorative treatment, the main factors implicated were related to individual socioeconomic position, however the city-level contextual effect should also be considered. Regarding need for tooth extraction, the contextual effect does not seem to be important and, in relation to the needs for prosthetic treatment, the final model showed effect of individual-level and city-level. Variables related to health services did not show significant effects.

**Conclusions:**

Dental treatment needs related to primary care (restoration and tooth extraction) and secondary care (prosthesis) were strongly associated with individual socioeconomic position, mainly income and education, in Brazilian adults. In addition to this individual effect, a city-level contextual effect, represented by HDI, was also observed for need for restorations and prosthesis, but not for tooth extractions. These findings have important implications for the health policy especially for financing and planning, since the distribution of oral health resources must consider the inequalities in availability and affordability of dental care for all.

## Background

Oral diseases such as tooth decay, tooth loss and periodontal disease are universally prevalent in adults and considered a major public health problem [1]. Oral conditions affected 3.9 billion people, and untreated caries in permanent teeth was the most prevalent condition evaluated for the entire Global Burden of Disease 2010 Study [2]. There are numerous studies on the social determinants and inequalities in oral health [3–7], but few on determinants of dental treatment needs in nationally representative samples.

The World Health Organization (WHO) has proposed criteria for assessing dental treatment needs in oral health epidemiological surveys [8]. In addition to the diagnosis of dental caries provided by the DMFT (number of teeth decayed, missing and filled), treatment needs record the extent and type of treatment needed for a given tooth. Thus, the existence of dental treatment needs is a measure of the absence or failure of oral health care and the difficulty of people to obtain a suitable and affordable dental service. Thus, as oral diseases, dental treatment needs are influenced by socioeconomic factors and are unequally distributed in populations.

In adults, the chronic cumulative nature of caries makes treatment needs a severe problem. Data from the recent national oral health survey in Brazil [9] show that adults aged 35–44 years have an average of 11 teeth needing any kind of clinical dental procedure and almost 70% needed a dental prosthesis to replace missing teeth.

The determinants of oral diseases are well explained by factors at the individual level. However, individual proximal factors are inadequate explanations of the prevalence and distribution among populations [10]. For example, tooth loss and caries are strongly associated with demographic factors, health related and psychological factors, socioeconomic status, use of health services and social capital [11, 12].

The use of multilevel statistical techniques has facilitated testing the effects of contextual level influences and their interactions with oral health [3]. Some studies found associations between contextual variables and dental caries experience [13, 14], as well as dental pain [15] and dental care needs in children [16].

Despite the fact that oral diseases occur in social contexts, there are few studies using multilevel analyses focusing on treatment needs. Thus, considering the importance of context in explaining oral health related inequalities, this study aims to evaluate the social determinants of dental treatment needs in 35–44 year old Brazilian adults, assessing whether inequalities in needs are expressed at individual and contextual levels.

## Methods

### Sources of data

Data on dental treatment needs were obtained from the national Brazilian Oral Health Survey in 2010, known as Project SBBrasil 2010. This household-based survey was conducted by the Brazilian Ministry of Health in 177 cities in the whole country, including the 27 state capitals. About 38,000 people divided into five age groups (5, 12, 15–19, 35–44 and 65–74 years-old) were interviewed and examined in their homes by trained and calibrated dentists, all workers of the Brazilian public health system. Further details about the sample design and other information have been reported elsewhere [17]. Socioeconomic indicators were obtained from different databases from National Demographic Census, carried out in 2010 by the Brazilian Institute of Geography and Statistics (IBGE). Data related to health system indicators were gathered from the health information systems of the Brazilian Ministry of Health.

### Variables

The dependent variables were based on the prevalence of normative dental treatment needs in adults. The classification of each subject was based on original variables available in databases and described below:

- **Prevalence of normative need for restorative treatment**. The criteria for assessing treatment needs for dental caries were based on the WHO criteria (1997). All teeth coded as needing “one surface filling” or “two or more surface fillings” were counted. Treatment need was categorized as “prevalence of at least 1 tooth with a need for restorative treatment”.
- **Prevalence of need for tooth extraction**. All teeth coded as needing “extraction” were counted. Based on its frequency distribution, the variable was categorized as “prevalence of at least 1 tooth needing tooth extraction”.
- **Prevalence of need for prosthetic treatment**. Represented by the percentages of people needing any kind of prosthesis, either in upper or lower jaws.

The first two variables represent oral health problems that can be treated by primary care services whereas in Brazil prosthetic treatment can only be carried out by specialized services (secondary care). Dental needs were based on a judgement made by a dental examiner who assessed the suitable treatment using specific criteria [9]. Needs are not a direct measure of oral disease, but may reflect caries severity and therefore are affected by the main determinants of dental caries. The prevalence of treatment needs also represents the failure of health system to provide sufficient and comprehensive oral health services. Each variable was considered as an outcome and it was analysed according its distribution in relation to socioeconomic variables.

The independent variables are related to economic status, education, demographic characteristics, infrastructure and sanitation conditions and health services availability. Table 1 presents all these variables, according to the level of analysis.

**Table 1:** **Independent variables according to the level of analysis**

Level	Variable	Type	Description
1^st^ (Individual)	Household Income	Economic	Total income received by all family members in the month preceding the survey, converted to Brazilian minimum wage
	Formal educational level	Education	Number of years of schooling counted from the first year of primary school
	Gender	Demographic	Sex of individual (male or female)
	Race	Demographic	Self-reported skin colour. From the five original categories, a dichotomous variable was created (white and black or mixed)
2^nd^ level (Census Sector)	Household income	Economic	Average income from residents aged 10 years or more. This age limit is considered to establish the Economically Active Population.
	Piped water	Sanitation	Percentage of households with piped water
	Garbage collection	Sanitation	Percentage of households with garbage collected from public or private companies
	Public lighting	Infrastructure	Percentage of households with available public lighting
	Paved streets	Infrastructure	Percentage of households with available paved streets
	Bathroom at home	House conditions	Percentage of households with at least one bathroom for exclusive use of residents
	Electricity	House conditions	Percentage of households with electricity
3^rd^ level (City)	Human Development Index (HDI)	Socioeconomic	Municipal Human Development Index. Geometric average of the indices *income*, *education* and *longevity*, with equal weights
	Oral Health Primary Care	Health Services	Percentage of population covered by oral health teams in the Family Health Programme
	Oral Health Secondary Care	Health Services	Rate of number of health units with specialized oral health services per 10,000 inhabitants

The five individual variables were obtained from a questionnaire administered to adults aged 35 to 44 years in their homes. This age group is recommended by the World Health Organization in oral health surveys to assess the oral health conditions in adults [8]. Household income and formal education level are representative of socioeconomic position, the former is about immediate conditions, whereas the latter is more related to life-course consequences of access to education. Sex and race are not used here as biological markers. Instead, they may reflect the socio-demographic position of these groups and how such position can affect distribution of oral diseases.

Socioeconomic indicators related to census sector were based on whether they expressed different social and environmental aspects at the neighbourhood level, such as the situation in relation to income, sanitation, infrastructure and house conditions.

The city-level socioeconomic variable related to human development was taken from the “Human Development Atlas” provided by the Brazilian agency of United Nations Development Program (http://www.pnud.org.br). The Human Development Index (HDI), a composite indicator that includes the level of education, longevity and income, is usually used by United Nations for comparisons of the level of quality of life in international basis. The same method used in worldwide countries was applied for all municipalities in Brazil. Indicators related to health services were obtained from the health information systems of Ministry of Health, the DATASUS system (http://www.datasus.gov.br). To assess the possible influence of health services at city level on the outcomes we followed the way these services are organized in Brazil. Essentially, public dental assistance is offered differently for primary and secondary care. The former is represented by the Family Health Programme, the main strategy of primary care in Brazil and the latter by the Centres of Specialized Dentistry, which are responsible for the more complex procedures, such as endodontic and surgical treatments. In the first case, an indicator was created from the percentage of the whole population covered by dental health services in primary care, the Family Health Programme (FHP). In the second case, to represent the coverage of oral health secondary care, it was calculated a rate of number of Centres of Specialized Dentistry per 10,000 inhabitants.

Considering the high number of variables in the second level and also taking into account that these variables belong to similar dimensions and furthermore present significant correlations, factor analysis was performed based on their principal components (PCA). Initially, the seven variables were evaluated in relation to correlations between them. All Pearson correlation values were significant and ranged between 0.30 and 0.90. The PCA identified two factors which explained 69% of the total variance. After Varimax rotation, the eigenvalues for these factors were calculated (Table 2). The value for Kaiser-Meyer-Olkin (KMO) was 0.839 indicating a good sampling adequacy and the Bartlett’s test of sphericity was significant (p < 0.001). Thus, two factors were created, representing the previous variables. Considering the characteristics of each grouped variable, the factor 1 was named as “income and infrastructure conditions” and the factor 2 was classified as “house conditions”. A single indicator based on the sum of the previous ones was created and named as “socioeconomic index”.

**Table 2:** **Rotated component matrix for the variables included in the factor analysis for census sector**

Census sector	Component	
Variable	1	2
Household income	0.538	
Piped water access	0.766	
Garbage collection	0.755	
Public lighting	0.875	
Paved streets	0.889	
Bathroom at home		0.807
Electricity		0.876

Principal Component Analysis was the extraction method and the rotation was Varimax with Kaiser Normalization.

### Statistical analysis

**Figure 1:**
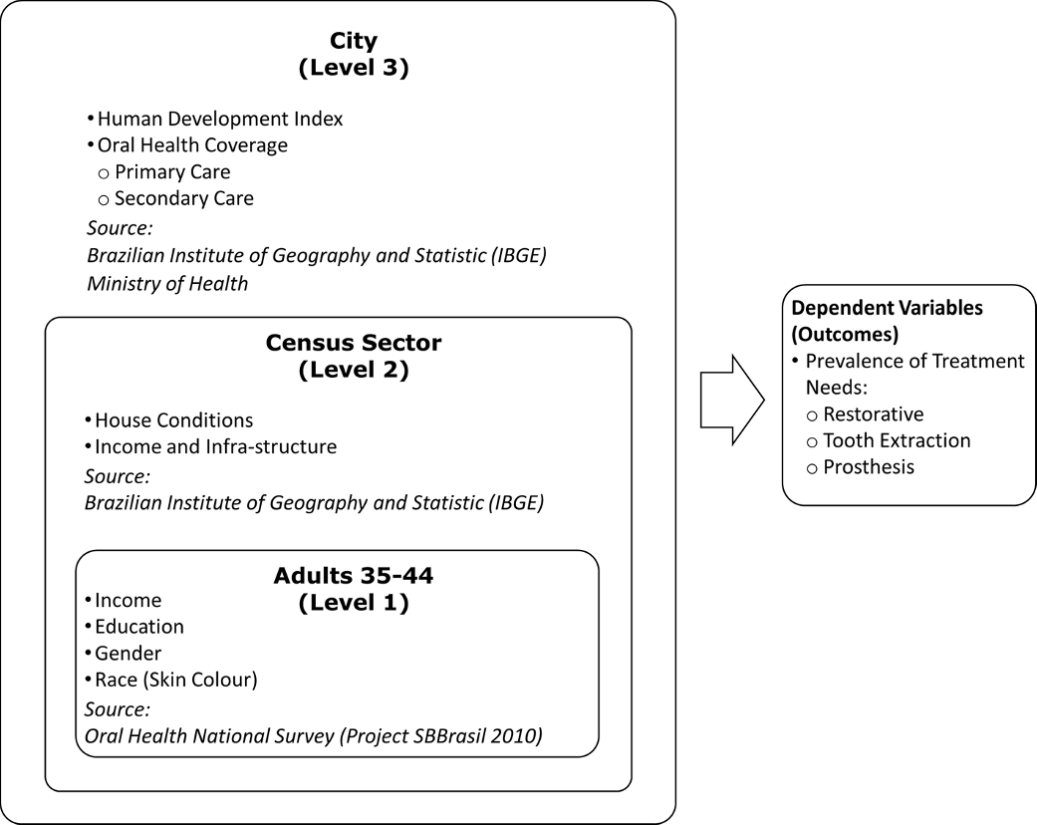
**Levels of analysis and respective variables and sources.**

A three-level multilevel mixed-effect Poisson regression analysis was performed to verify the effect of individual characteristics and also the context’s influence on the distribution of outcomes. In this case, the context was represented by two hierarchical levels of aggregation, taking into account the Brazilian administrative organization (Figure 1). Individuals were nested in “census sectors”, an area limited by streets, with an average of 300 households inside. The criteria to group these domiciles within sectors are usually based on socioeconomic characteristics and it was performed by IBGE. The estimated number of census sectors in Brazil is 310,000. This level represents the neighbourhood contextual effect. Finally, census sector were nested in cities, the third level. Brazil has 5,665 cities in 27 federative units. Thus, in the oral health database, there are data from 9,564 individuals aged 35–44 years old, nested in 1,105 census sectors nested in 177 cities (27 state capitals plus 150 from countryside).

In order to assess whether there was a gradient in the distribution of outcomes in relation to the factors, some variables were categorized in a three or four-level ordinal variable based on their distribution.

Exploratory analysis was performed evaluating the effect of each level through calculating Prevalence Ratios (PR) with respective 95% confidence intervals with the better situation as the reference category. In addition, a three-level multilevel modelling was constructed for all outcomes and significant explanatory variables. The analysis started with a random intercepts model (null model), in order to verify whether the contextual effects were significant. This was done by observation of between-city and between-neighbourhood variances and also by the Likelihood Ratio (LR) test. The predictor variables were added to the null model and the final model was achieved when variables were added and the model remained significant.

### Ethical issues

This study was based on secondary data obtained from several publicly available datasets, therefore did not require ethical approval.

## Results

From the whole survey database 9,564 records were extracted from adults aged 35–44 years. There were no missing values for the variables sex, HDI and oral health services in secondary care. Household income had 227 (2.37%) missing values, educational level had 69 (0.72%) and race had 233 (2.44%). For the second level, there were 85 (0.89%) missing values and for oral health services in primary care, 265 (2.77%). As no variable had more than 3% of missing values, we did not perform data imputation.

Table 3 shows the results for the descriptive analysis of the dependent variables in relation to the explanatory variables. At individual level, there was a typical social gradient for all outcomes in relation to income and education. About 70% of low-income subjects had at least one tooth needing restorative treatment compared to 38% in those earning 5 and more minimum wages. The prevalence of need for tooth extraction also showed a gradient with a larger gap; from 25% in low-income category to about 6% in the better-off group. Need for prosthetic treatment varied from 47% in the most affluent group to nearly 85% in the poorest group. All outcomes were also related to race. For both explanatory variables the gap was more pronounced in relation to the need for tooth extraction. Sex affected significantly the distribution of outcomes but with an upper limit of the CI 95% quite close to 1.

**Table 3:** **Bivariate associations between outcomes and the independent variables according to the levels**

		Treatment needs					
		Restorative		Extraction		Prosthesis	
	n	% (CI 95%)	PR (CI 95%)	% (CI 95%)	PR (CI 95%)	% (CI 95%)	PR (CI 95%)
Individual level							
*Household Income*							
5 and more MW	1,441	38.0 (35.4;40.5)	Ref	5.8 (4.5;7.0)	Ref	47.0 (44.4;49.5)	Ref
3 to 5 MW	1,805	51.4 (49.0;53.7)	1.35 (1.25;1.46)	11.4 (9.9;12.8)	1.97 (1.54;2.51)	67.8 (65.6;69.9)	1.44 (1.35;1.53)
1 to 3 MW	4,687	62.7 (61.3;64.0)	1.65 (1.54;1.76)	18.9 (17.7;20.0)	3.27 (2.63;4.08)	79.9 (78.7;81.0)	1.70 (1.60;1.80)
Up to 1 MW	1,404	70.6 (68.2;72.9)	1.85 (1.72;2.00)	25.4 (23.1;27.6)	4.40 (3.52;5.55)	84.5 (82.6;86.3)	1.79 (1.69;1.90)
*Education (years of schooling)*							
12 and more	2,226	43.6 (41.5;45.6)	Ref	8.4 (7.2;9.5)	Ref	54.0 (51.9;56)	Ref
9 to 11	2,963	57.0 (55.2;58.7)	1.30 (1.23;1.38)	13.7 (12.4;14.9)	1.63 (1.38;1.92)	73.7 (72.1;75.2)	1.36 (1.30;1.42)
6 to 8	1,961	64.6 (62.4;66.7)	1.48 (1.39;1.56)	19.6 (17.8;21.3)	2.34 (1.98;2.76)	80.6 (78.8;82.3)	1.49 (1.42;1.56)
Up to 5	2,345	66.6 (64.6;68.5)	1.52 (1.44;1.61)	24.9 (23.1;26.6)	2.97 (2.55;3.48)	84.9 (83.4;86.3)	1.57 (1.50;1.64)
*Sex*							
Male	3,277	61.2 (59.5;62.8)	Ref	18.2 (16.8;19.5)	Ref	72.7 (71.1;74.2)	Ref
Female	6,287	56.1 (54.8;57.3)	0.91 (0.88;0.95)	15.5 (14.6;16.3)	0.85 (0.77;0.93)	73.6 (72.5;74.6)	1.01 (0.98;1.03)
*Race (Skin colour)*							
White	4,049	50.2 (48.6;51.7)	Ref	12.6 (11.5;13.6)	Ref	64.4 (62.9;65.8)	Ref
Black or Mixed	5,282	63.4 (62.1;64.6)	1.26 (1.21;1.31)	19.2 (18.1;20.2)	1.52 (1.38;1.68)	80.0 (78.9;81.0)	1.24 (1.21;1.27)
Neighbourhood level							
*Socioeconomic Index*							
3^rd^ Tertile (Better-off)	3,163	48.9 (47.1;50.6)	Ref	12.2 (11;13.3)	Ref	62.4 (60.7;64.0)	Ref
2^nd^ Tertile	3,143	61.3 (59.5;63.0)	1.25 (1.20;1.31)	17.3 (15.9;18.6)	1.42 (1.26;1.60)	78.0 (76.5;79.4)	1.25 (1.21;1.29)
1^st^ Tertile (Worst)	3,173	63.8 (62.1;65.4)	1.30 (1.25;1.36)	19.8 (18.4;21.1)	1.62 (1.44;1.83)	79.7 (78.2;81.1)	1.28 (1.24;1.32)
City Level							
*Human Development Index*							
0.79 and more (Better-off)	3,075	48.5 (46.7;50.2)	Ref	13.6 (12.3;14.8)	Ref	64.6 (62.9;66.2)	Ref
0.74 to 0.78	2,867	59.9 (58.1;61.6)	1.23 (1.18;1.29)	18.3 (16.8;19.7)	1.34 (1.19;1.51)	75.6 (74.0;77.1)	1.17 (1.13;1.21)
Up to 0.74 (Worst)	3,622	64.2 (62.6;65.7)	1.32 (1.27;1.38)	17.3 (16.0;18.5)	1.27 (1.13;1.42)	78.8 (77.4;80.1)	1.22 (1.18;1.26)
*Oral Health Services in Primary Care*							
3^rd^ Tertile (Better-off)	3,099	57.4 (55.6;59.1)	Ref	15.9 (14.6;17.1)	Ref	73.6 (72.0;75.1)	Ref
2^nd^ Tertile	2,988	57.9 (56.1;59.6)	1.01 (0.97;1.05)	16.1 (14.7;17.4)	1.01 (0.9;1.14)	73.3 (71.7;74.8)	1.00 (0.97;1.03)
1^st^ Tertile (Worst)	3,212	58.5 (56.7;60.2)	1.02 (0.98;1.06)	17.2 (15.8;18.5)	1.08 (0.97;1.21)	73.7 (72.1;75.2)	1.00 (0.97;1.03)
*Oral Health Services in Secondary Care*							
3^rd^ Tertile (Better-off)	3,188	53.3 (51.5;55.0)	Ref	15.8 (14.5;17.0)	Ref	72.1 (70.5;73.6)	Ref
2^nd^ Tertile	2,893	62.5 (60.7;64.2)	1.17 (1.12;1.22)	18.0 (16.6;19.3)	1.14 (1.02;1.28)	73.1 (71.4;74.7)	1.01 (0.98;1.04)
1^st^ Tertile (Worst)	3,483	58.2 (56.5;59.8)	1.09 (1.05;1.14)	15.7 (14.4;16.9)	0.99 (0.89;1.11)	74.6 (73.1;76.0)	1.03 (1.00;1.06)

MW = Minimum Wage; CI = Confidence Interval; PR = Prevalence Ratio.

Regarding the analysis of the second level, there were significant differences by neighbourhood socioeconomic index for all outcomes. People living in socially-deprived areas had about 30% more restorative and prosthetic needs and about 60% more need for tooth extraction. The city-level analysis, considering the Human Development Index, showed a similar pattern. Regarding both health services indicators, no differences were found for all outcomes. An impressive equal distribution was observed in relation to oral health coverage in primary and secondary care, with the results being practically the same in all categories.

All significant effects were fitted into a three-level multilevel analysis with mixed effects. Preliminarily, all outcomes were fitted in a null model in order to verify the contextual effects (Table 4). As we were working with two contextual levels (city and neighbourhood), their effects were tested separately and, thereafter, both levels were included in the model. In relation to the need for restorative treatment, the between-city and the between-neighbourhood variance were estimated as 0.029 and 0.012 respectively. Either considering each level separately or including both, the Likelihood Ratio (LR) test showed a significant effect. Similarly, the need for extraction also show significant results for the contextual effects (p < 0.001). Regarding need for prosthesis, the between-neighbourhood variance was zero and hence the LR test was not significant (p = 1.00). However, city level was significant (p < 0.001). Based on these results, we performed a multilevel analysis including both contextual levels for the need for restorative treatment and for the need for extraction. For the need for prosthetic treatment, only the city-level was included.

**Table 4:** **Fixed and random effects parameters in the multilevel mixed-effect Poisson regression analysis for the null model according to outcomes**

	Treatment needs					
	Restorative		Extraction		Prosthesis	
Fixed Effects	Intercept	95% CI	Intercept	95% CI	Intercept	95% CI
City Level	−0.56	−0.61;-0.51	−1.90	−1.95;-1.78	−0.31	−0.34;-0.27
Neighbourhood Level	−0.55	−0.58;-0.52	−1.90	−1.96;-1.83	−0.30	−0.32;-0.28
Both	−0.56	−0.61;-0.51	−1.92	−2.01;-1.83	−0.31	−0.34;-0.27
Random Effects	Variance (SE)	LR Test (Chi^2^; p)	Variance (SE)	LR Test (Chi^2^; p)	Variance (SE)	LR Test (Chi^2^; p)
City level only	0.029 (0.008)	88.7; <0.001	0.068 (0.026)	37.82; <0.001	0.013 (0.004)	50.16; <0.001
Neighbourhood Level only	0.012 (0.006)	5.30; 0.011	0.169 (0.035)	41.25; <0.001	0.000 (0.000)	0.00; 1.000
Both		88.7; <0.001		62.47; <0.001		
City level	0.029 (0.008)		0.056 (0.023)			
Neighbourhood Level	0.000 (0.000)		0.128 (0.032)			

CI = Confidence Interval; LR = Likelihood Ratio.

**Figure 2:**
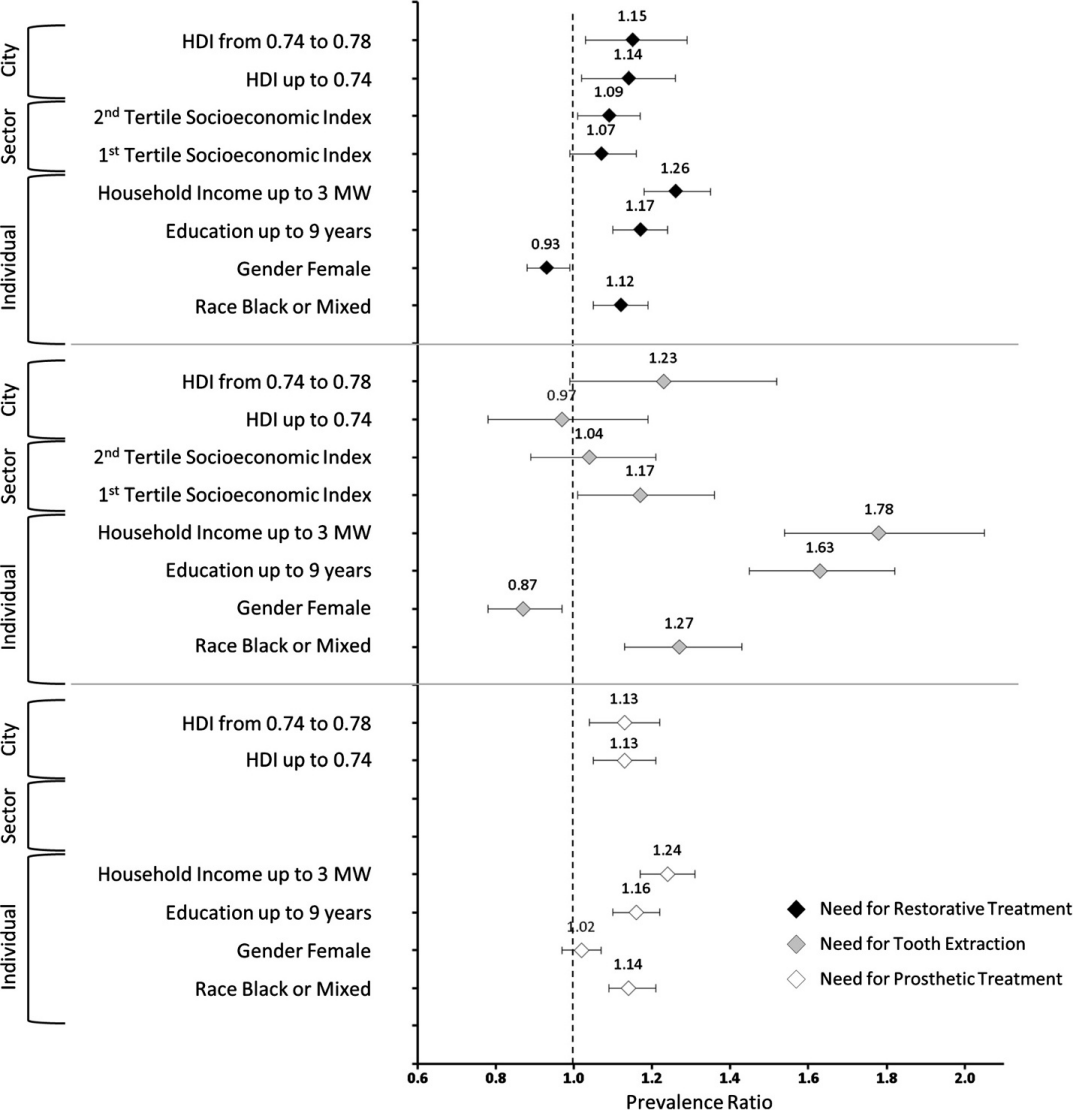
**Adjusted Prevalence Ratios and respective Confidence Intervals (95%) for the outcomes according to the independent variables and levels of analysis.**

Table 5 shows results for these models, according to the three outcomes. In relation to the need for restorative treatment, when the other levels were included into the analysis, most of variables remained significant with a slight adjustment in the prevalence ratios (PR). The city-level variance dropped from 0.029 to 0.011 (62%) indicating a large between-city effect. Moreover, the p-value for the LR test in model 2 indicates that it is the most appropriate to describe the individual and contextual effects. The effect of neighbourhood almost disappeared, remaining significant only in the worst tertile of Socioeconomic Indicator. Comparing the values of PR in the bivariate analysis, the adjustment was higher in HDI, reducing its effect. Thus, in relation to the need for restorative treatment, the main factors implicated are related to individual socioeconomic position, however the city-level contextual effect should also be considered.Regarding need for tooth extraction, there was a strong effect initially in relation to the neighbourhood level that almost disappeared in the final model and the HDI also became non-significant. Therefore, the contextual effect does not seem to be important for this outcome. On the other hand, the level of inequality in relation to individual socioeconomic indicators is the strongest of all outcomes (Figure 2). Compared to the better-off group, about 80% more people with income up to 3 times the minimum wage needed extractions, after controlling for all other variables.Finally, in relation to the needs for prosthetic treatment, the final model (model 2) showed effect of individual-level variables (income, education and race) and city-level (HDI) since the neighbourhood level was not included. The magnitude of the effects, observed by the comparison of PRs, were similar to the effects observed for need for restorative treatment (Figure 2).

**Table 5:** **Multilevel mixed-effect poisson regression analysis for the three outcomes**

	Treatment needs											
	Restorative				Extraction				Prosthesis			
	Model 1 (n = 9,061)		Model 2 (n = 8,977)		Model 1 (n = 9,061)		Model 2 (n = 8,977)		Model 1 (n = 9,061)		Model 2 (n = 8,977)	
City Level	PR (95% CI)	p-value	PR (95% CI)	p-value	PR (95% CI)	p-value	PR (95% CI)	p-value	PR (95% CI)	p-value	PR (95% CI)	p-value
HDI 0.74 a 0.78			1.15 (1.03;1.29)	0.011			0.97 (0.78;1.19)	0.809			1.13 (1.04;1.22)	0.002
HDI up to 0.74 (Worst)			1.14 (1.02;1.26)	0.015			1.23 (0.99;1.52)	0.060			1.13 (1.05;1.21)	0.003
Neighbourhood Level												
SEI 2^nd^ Tertile			1.07 (0.99;1.16)	0.059			1.04 (0.89;1.21)	0.614			-	-
SEI 1^st^ Tertile (Worst)			1.09 (1.01;1.17)	0.031			1.17 (1.01;1.36)	0.041			-	-
Individual Level												
Household Income up to 3 MW	1.29 (1.21;1.38)	<0.001	1.26 (1.18;1.35)	<0.001	1.85 (1.61;2.12)	<0.001	1.78 (1.54;2.05)	<0.001	1.24 (1.18;1.32)	<0.001	1.24 (1.17;1.31)	<0.001
Up to 9 years of schooling	1.18 (1.11;1.25)	<0.001	1.17 (1.10;1.24)	<0.001	1.63 (1.46;1.82)	<0.001	1.63 (1.45;1.82)	<0.001	1.16 (1.10;1.22)	<0.001	1.16 (1.10;1.22)	<0.001
Female	0.93 (0.88;0.99)	0.017	0.93 (0.88;0.99)	0.021	0.88 (0.79;0.97)	0.016	0.87 (0.78;0.97)	0.010	1.02 (0.97;1.07)	0.512	1.02 (0.97;1.07)	0.464
Black or Mixed	1.13 (1.07;1.20)	<0.001	1.12 (1.05;1.19)	<0.001	1.28 (1.14;1.43)	<0.001	1.27 (1.13;1.43)	<0.001	1.15 (1.09;1.21)	<0.001	1.14 (1.09;1.21)	<0.001
Fixed Effects												
Intercept (95% CI)	−0.56 (−0.61;-0.51)		−0.98 (−1.09;-0.88)		−2.69 (−2.85;-2.52)		−2.76 (−2.98;-2.54)		−0.63 (−0.70;-0.56)		−0.71 (−0.79;-0.63)	
Random Effects	Variance (SE)		Variance (SE)		Variance (SE)		Variance (SE)		Variance (SE)		Variance (SE)	
City level	0.019 (0.006)		0.011 (0.005)		0.049 (0.020)		0.042 (0.019)		0.007 (0.003)		0.004 (0.002)	
Neighbourhood Level	0.000 (0.000)		0.000 (0.000)		0.035 (0.027)		0.033 (0.027)		-		-	
LR Test (Chi^2^; p-value)	23.38; <0.001		20.12; <0.001		29.05; <0.001		23.24; <0.001		14.92; 0.001		5.34; 0.010	

PR = Prevalence Ratio; CI = Confidence Interval; HDI = Human Development Index; SEI = Socioeconomic Index; MW = Minimum Wage; LR = Likelihood Ratio. The significant values are in bold.

Model 1 for the individual variables only, model 2 for individual + city (prosthesis) or for the three levels (restorative and extraction).

## Discussion

This study assessed the individual and contextual determinants of dental treatment needs in Brazilian adults. The indicators used, namely the prevalence of treatment needs for dental fillings, tooth extraction and dental prosthesis represent essentially graded levels of access to oral health services as well as the quality of such services. Despite the gradient observed in relation to the socioeconomic conditions, the prevalence of these treatment needs was high. About 58% of Brazilian adults have one or more teeth needing restorative procedures; 16% have at least one tooth indicated for extraction and 73% needed to replace missing teeth with prosthesis. Considering the population estimated for 2010, these figures represent respectively 15.6, 4.3 and 19.6 million of people in this age group. There are few comparable studies that used similar indicators. Most of the cross-sectional surveys in adult populations report high levels of both caries experience and untreated teeth. In UK, almost a third of the dentate adults had visible caries into the dentine, “representing many millions of people with decay” [18]. In Canada, Ramraj et al. [19] reported a prevalence of 35% of at least one clinically determined treatment need in adults aged 40–59 years.

In the present study, the distribution of the three outcomes was highly influenced by the socioeconomic position at individual level, which remained significant even after accounting for contextual effects. For the need for tooth extraction, individual socioeconomic position was the main effect observed since the city level influence was not significant and the neighbourhood level showed a small effect only for the worst socioeconomic tertile. In addition, the need for tooth extraction had the strongest relationship with socioeconomic position, with the highest prevalence ratios. In other words, irrespective of the contextual situation, being a man with skin colour black or mixed, earning up to the equivalent to one minimum wage and having studied for only five years, appears to be the best predictor of need for tooth extraction.

As the dependent variables in this study are related to treatment needs that could be treated by primary and secondary dental care, the main effect of the context would essentially be an increase of opportunities to achieve such services [3, 10]. Apparently, in Brazil, this was the case for restorative treatment need and prosthetic treatment need, but not for the need for tooth extraction. In fact, although a reduction in dental services utilization disparities has been reported in Brazil in recent years [20], the capacity to access dental services is still strongly modulated by socioeconomic position as dental services utilization is often associated with socioeconomic variables [20, 21]. About 75% of the Brazilian families earn up to five times the minimum wage [22]. All these people are potentially dependent on the public health system for dental care, as they cannot afford private treatment. Furthermore, the distribution of health workforce, dental workers included, is unequal [23] and has increased since the 1990’s [24]. Thus, poor Brazilian adults are likely to have more teeth extracted; a situation strongly influenced by their income and educational level.

Regarding the need for restorative and prosthetic treatment, the individual effect remained significant but there was also a contextual effect of city level. In both cases, living in cities with better quality of life, namely, a higher HDI, meant having lower dental treatment needs. It is important to stress that this is not an attenuating effect of the context. On the contrary, it reproduces, at a city level, the inequality observed at the individual level. When the effects of individual socioeconomic position were fractioned for the highest and lowest HDI (data not displayed), the prevalence ratios remained the same. That suggests that policies aimed at tackling these inequalities should focus on determinants at both the individual and city (area) levels.

The different results in relation to the contextual effect for the different outcomes are not easily explained because there are no comparable studies on treatment needs in the literature. Taking into account other studies with different indicators and age groups, the contextual effect was either at national or local levels. At the local level, there was a contextual effect at city and/or neighbourhood level on dental status [10], dental pain [15, 25] and dental caries [3, 14, 26, 27]. On a national basis, Celeste et al. [28] found that greater municipal income inequality was associated with poorer oral health in Brazilian adults, after controlling for variables at individual level. Using the same dataset, Antunes et al. [16] also found a significant city-level effect of HDI on the DMFT in 12-years-old children.

Thus, the contextual effect, as a determinant of the distribution of health and illness, whether at neighbourhood, census tracks, cities or countries levels, cannot be ruled out. However, there is no general explanation which could be applied for all cases. Instead, a singular theory must be constructed depending on the nature of the indicators used and the explanatory variables. In our study the influence of context could be possibly related to the opportunities to gain access to oral health services for the simplest to the more complex dental treatment levels. However, our results do not strongly support such an explanation as cities with low coverage of both primary and secondary dental health services had levels of treatment needs that were similar to those cities with better dental services coverage. We previously argued that a significant effect of a contextual variable, such as HDI, controlled by the individual socioeconomic position, could mean that health inequality is expressed at city level. In the case of variables related to dental health services, the interpretation could be that there is no inequality in distribution of dental health resources. Obviously, a most desirable effect would be an inverse association, with more services being deployed in those regions with the worst dental situation. However, a direct association was not found with dental needs; a typical case of Hart’s “inverse care law” [29]. While there is not much evidence of the importance of contextual factors on our findings, it is also the case that we are limited by data availability. Water fluoridation would potentially be an important determinant at contextual level, with 40% of Brazilians drinking fluoridated water. However, the role of water fluoridation could not be explored in our analyses due to the lack of available data at neighbourhood level in terms of the percentage of the population covered.

There are some strengths and limitations of this study. A strength is the comprehensiveness of the survey from which the data were extracted. The sample size also permits the use of reliable population estimates as well as prevalence ratios with narrow confidence intervals. In addition, the independent variables at city and neighbourhood levels were extracted from national official databases from population census. On the other hand, as the dependent variables are based on the clinical judgment of several dental examiners, they are susceptible to bias. Although the criteria are very clear and the training and calibration of the teams were well done, some subjectivity in the judgment and hence some disagreement may have occurred.

The main results reported here have important implications for dental public health. A core principle of the Brazilian National Health System states that the distribution of health services must be based on equity, which means a positive discrimination in terms of priorities. The existence of inequalities in the need for restorative and prosthetic treatment expressed in both individual and city levels calls for more efforts to reorganize the model of health financing.

## Conclusions

Dental treatment needs related to primary care (restoration and tooth extraction) and secondary care (prosthesis) were strongly associated with individual socioeconomic position, mainly income and education, in Brazilian adults. In addition to this individual effect, a city-level contextual effect, represented by HDI, was also observed for need for restorations and prosthesis, but not for tooth extractions. These findings have important implications for the health policy especially for financing and planning, since the distribution of oral health resources must consider the inequalities in availability and affordability of dental care for all.
